# Plant-derived extracellular vesicles (PDEVs) in nanomedicine for human disease and therapeutic modalities

**DOI:** 10.1186/s12951-023-01858-7

**Published:** 2023-03-29

**Authors:** Zhijie Xu, Yuzhen Xu, Kui Zhang, Yuanhong Liu, Qiuju Liang, Abhimanyu Thakur, Wei Liu, Yuanliang Yan

**Affiliations:** 1 Department of Pathology, Xiangya Hospital, Central South University, Changsha, Hunan 410008 China; 2 Department of Rehabilitation, The Second Affiliated Hospital of Shandong First Medical University, Taian, Shandong 271000 China; 3 State Key Laboratory of Silkworm Genome Biology, Medical Research Institute, Southwest University, Chongqing, 400715 China; 4 Department of Pharmacy, Xiangya Hospital, Central South University, Changsha, Hunan 410008 China; 5 Pritzker School of Molecular Engineering, Ben May Department for Cancer Research, University of Chicago, Chicago, IL 60637 USA; 6 Department of Orthopedic Surgery, The Second Hospital University of South China, Hengyang, Hunan 421001 China; 7 National Clinical Research Center for Geriatric Disorders, Xiangya Hospital, Central South University, Changsha, Hunan 410008 China

**Keywords:** Plant-derived extracellular vesicles, Nanomedicine, Cell-cell communication, Human diseases, Cancer

## Abstract

**Background:**

The past few years have witnessed a significant increase in research related to plant-derived extracellular vesicles (PDEVs) in biological and medical applications. Using biochemical technologies, multiple independent groups have demonstrated the important roles of PDEVs as potential mediators involved in cell-cell communication and the exchange of bio-information between species. Recently, several contents have been well identified in PDEVs, including nucleic acids, proteins, lipids, and other active substances. These cargoes carried by PDEVs could be transferred into recipient cells and remarkably influence their biological behaviors associated with human diseases, such as cancers and inflammatory diseases.

**Main body of the abstract:**

This review summarizes the latest updates regarding PDEVs and focuses on its important role in nanomedicine applications, as well as the potential of PDEVs as drug delivery strategies to develop diagnostic and therapeutic agents for the clinical management of diseases, especially like cancers.

**Conclusion:**

Considering its unique advantages, especially high stability, intrinsic bioactivity and easy absorption, further elaboration on molecular mechanisms and biological factors driving the function of PDEVs will provide new horizons for the treatment of human disease.

## Background

Although the beneficial properties of natural substances against human diseases have been recognized for several decades, clarification of the biological functions and underlying molecular mechanisms remains limited. In recent years, the use of extracellular vesicles (EVs) from natural compounds has gained huge scientific interest as a promising therapeutic strategy [1]. These nanometer-sized membrane-enclosed EVs have been extracted from many plant species [2], such as *Dendropanax morbifera* [3], grapefruit [4], and dried plant-derived materials [5]. These EVs are effectively uptaken by most host organs, affecting their physiological and pathological processes [4]. Nowadays, several studies have pointed out the important roles of plant-derived extracellular vesicles (PDEVs) in cell-cell communication, the exchange of bio-information between different cells, and maintaining proper tissue homeostasis and organism integrity [6, 7]. The biochemical and pharmacological studies have demonstrated the heterogeneity of PDEVs in terms of their origin, size, and content [8]. Generally, according to the size, the PDEVs are mainly divided into two categories: large microvesicles (100 to 1,000 nm) and small nanovesicles (50 to 100 nm) [9, 10].

In the 1960s, the identification of PDEVs was first attributed to Jensen’s group, who found that the multivesicular bodies were released from cotton cells [11] and carrot cells [12]. They observed that the multivesicular bodies could be released into extracellular space by the membrane fusion reaction. It was not until 2009 that Regente et al. [13] isolated the EVs from sunflower apoplastic fluids and demonstrated the presence of some proteins in these PDEVs. More importantly, in 2017, the evidence from the study by Rutter and Innes [14] indicated the promising biological activity of leaf apoplast-derived EVs in the cellular defense system. Subsequently, clarifying the biological roles and clinical applications of PDEVs has rapidly become an attractive research field [15]. Recent advances in PDEVs have demonstrated their several bioactivities, such as anti-cancer, anti-inflammatory, and anti-oxidative stress properties, in vitro and in vivo [16]. Despite this, more studies need to be performed to explore the underlying molecular mechanisms behind recipient cellular targets regulated by PDEVs. In addition, our current knowledge is not sufficient to distinguish the different biological activities and therapeutic efficacy of PDEVs from different kinds of plants.

Although the sources of PDEVs are distinct, they might have promising aspects against pathological conditions (Table 1). In this review, we mainly summarize the up-to-date pre-clinical and clinical studies to discuss the potential of PDEVs in clinical applications and therapeutic implications. This comprehensive review proposes to focus on the characterization and biomedicinal applications of PDEVs to improve human health.

**Table 1:** Summary of PDEVs in multiple pathological conditions

Plant species	Molecular mechanism	Biological function	Diseases	Refs
*Kaempferia parviflora*		Suppression of cell viability	Gastric cancer	(60)
Tea flowers/leaves	Stimulating ROS generation	Anti-proliferation, anti-migration	Breast cancer	(61, 62)
Lemon	Improving ROS concentration	Induction of cell apoptosis	Gastric cancer	(64)
Bitter melon		Sensitizing effect of 5-fluorouracil	Oral squamous cell carcinoma	(68, 69)
*Petasites japonicus*	Activating MAPK and NF-κB signaling	Promoting maturation of dendritic cells, activating Th1/cytotoxic T cells	SARS-CoV-2 infection	(81)
*Pueraria lobata*	Facilitating M2 macrophage polarization	Anti-inflammatory	Inflammatory-related diseases	(82)
Oat	Decreasing secretion of pro-inflammatory cytokines	Prevention of ethanol-induced brain damage	Brain damage	(84)
Lemon	Inhibiting ERK/NF-κB signalling	Anti-inflammatory effects	Inflammatory damage	(85)
Carrot	Up-regulating antioxidative molecules	Alleviating oxidative stress	Myocardial infarction	(90)
*Aloe saponaria*		Promoting tube formation and angiogenesis	Skin wound	(96)
Ginseng	Delivery of plant microRNAs into BMSCs	Facilitating BMSC neural differentiation and neural restoration	Skin wound	(97)
Ginseng	Altering polarization of M2 macrophages	Improving anti-tumor immune response	Melanoma	(100)
*Dendropanax morbifera*	Impairing tyrosinase-related signaling	Reducing melanin concentration and increased whitening effect	Melanoma	(101)

### Comparing PDEVs to mammalian EVs

For a long time, the EVs from animals, mainly mammals, have been gradually used in genetic, biochemical, and pharmacological fields [17–20]. In 1987, the first report of mammalian EVs was that exosomes were derived from the maturing reticulocyte [21]. After then, a large number of EVs have been constitutively identified from almost all of the mammalian cells, such as cancer cells [22, 23], immune cells [24] stem cells [25], etc. In addition, mammalian EVs have also been discovered in the biological fluids, such as urine, blood and saliva [26, 27]. Mammalian EVs contain diverse cargo molecules (proteins/nucleic acids/lipids) and could be involved in the intercellular communication [28]. Because of their satisfactory pharmacological activity and biocompatibility, mammalian EVs are widely regarded as the regulatory agents in multiple physiological and pathological processes. In myocardial infarction mice model, injection of dendritic cell-derived exosomes could promote the infiltration of Treg cells and M2 macrophages into border zoom, consequently improving the cardiac function [29]. The exosomes derived from gefitinib sensitive cancer cells could effectively reverses the gefitinib resistance by transferring microRNA-7 in non-small-cell lung cancer [30]. Besides, Cui’s group further confirmed that mammalian exosomes could transport the bioactive molecules across the cellular interface, such as blood-brain barrier (BBB), and targeting glioma cells [31]. Although the biological application of mammalian EVs is promising, there are several major issues that pose the obstacles for their clinical translation, for example low yield, difficulties to obtain high-quality EVs, and time-consuming isolation [10]. In particular, the utilization of animals as a source of vesicles frequently activates the host immune responses, likely causing side-effects [23, 32]. However, identifying PDEVs and clarifying their functional mechanisms could represent an alternative strategy to overcome these challenges.

**Fig. 1:**
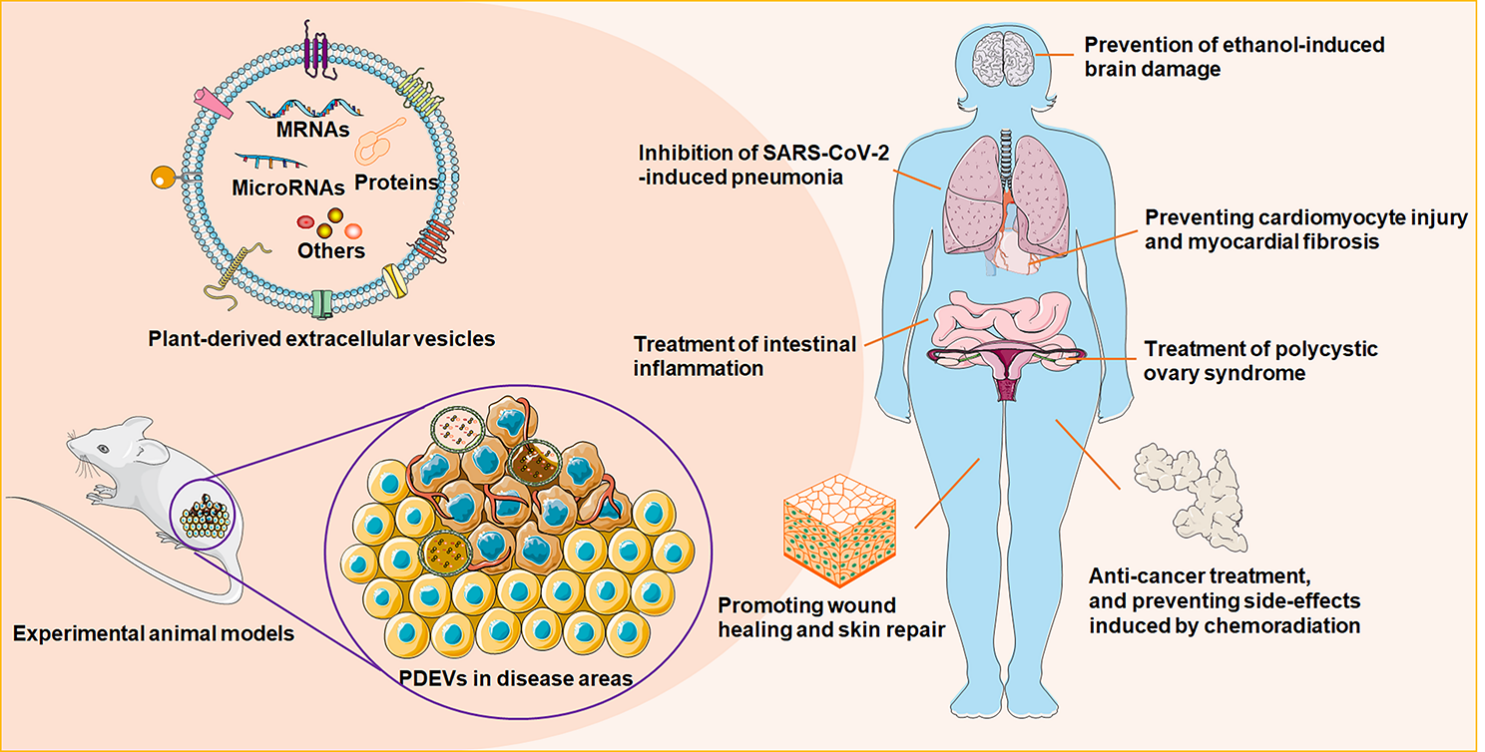
Application of plant-derived extracellular vesicles (PDEVs) as functional nanomedicine materials for human diseases

Recently, several research groups have speculated the existence of nanovesicles in plant materials, participating in cell-to-cell communication and interspecies communication [33]. The cargoes carried by PDEVs could be transferred into the receiving cells, causing changes in the (patho)physiological functions [34] (Fig. 1). Even though the secretion mechanisms of EVs from plant or animal cells are similar to some extent, such as exocytosis-mediated release [35], a few different biological characteristics can be identified among these EVs from different species. PDEVs can be obtained in large quantities due to the low cost of various plant resources. The plant-derived nanovesicles can be produced continuously from all kinds of fruits and vegetables purchased from conveniently located local markets [15]. Moreover, the plant cells in in vitro culture media could even be used to produce sufficient PDEVs [36]. Under controlled artificial conditions, the application of food additives successfully increased PDEV production [36]. They have no significant toxicity as they are mainly extracted from naturally medicinal or edible plant materials. Additionally, PDEVs are suitable for human health management without activating the host immune responses [37]. Besides, through several proposed mechanisms, including endocytosis, phagocytosis, macropinocytosis, and membrane fusion [38], PDEVs could easily pass across different biological barriers, such as the BBB, and are subsequently absorbed by the recipient cells [39]. Considering these benefits (Table 2), we need to dedicate more in-depth investigation to better clarify the basic characteristics of PDEVs as diagnostic and therapeutic agents, which will help open new avenues for the regulation of human health.

**Table 2:** The preliminary research about the benefits of plant-derived EVs

Items	Characteristics	Refs
Source	Almost all kinds of edible fruits and vegetables	(15)
Yield	Large production, and can be increased by food additives	(36)
Side-effects	No significant side-effects	(37)
Barrier crossing ability	Crossing the different biological barriers	(39)
Targeting	Carrying cargoes, and easily absorbed by the targeted recipient cells	(40)
Stability	Good stability in microenvironment	(60)
Cost	Low costs and easy of purchase	(103)

### Main pharmacological activities of PDEVs

**Fig. 2:**
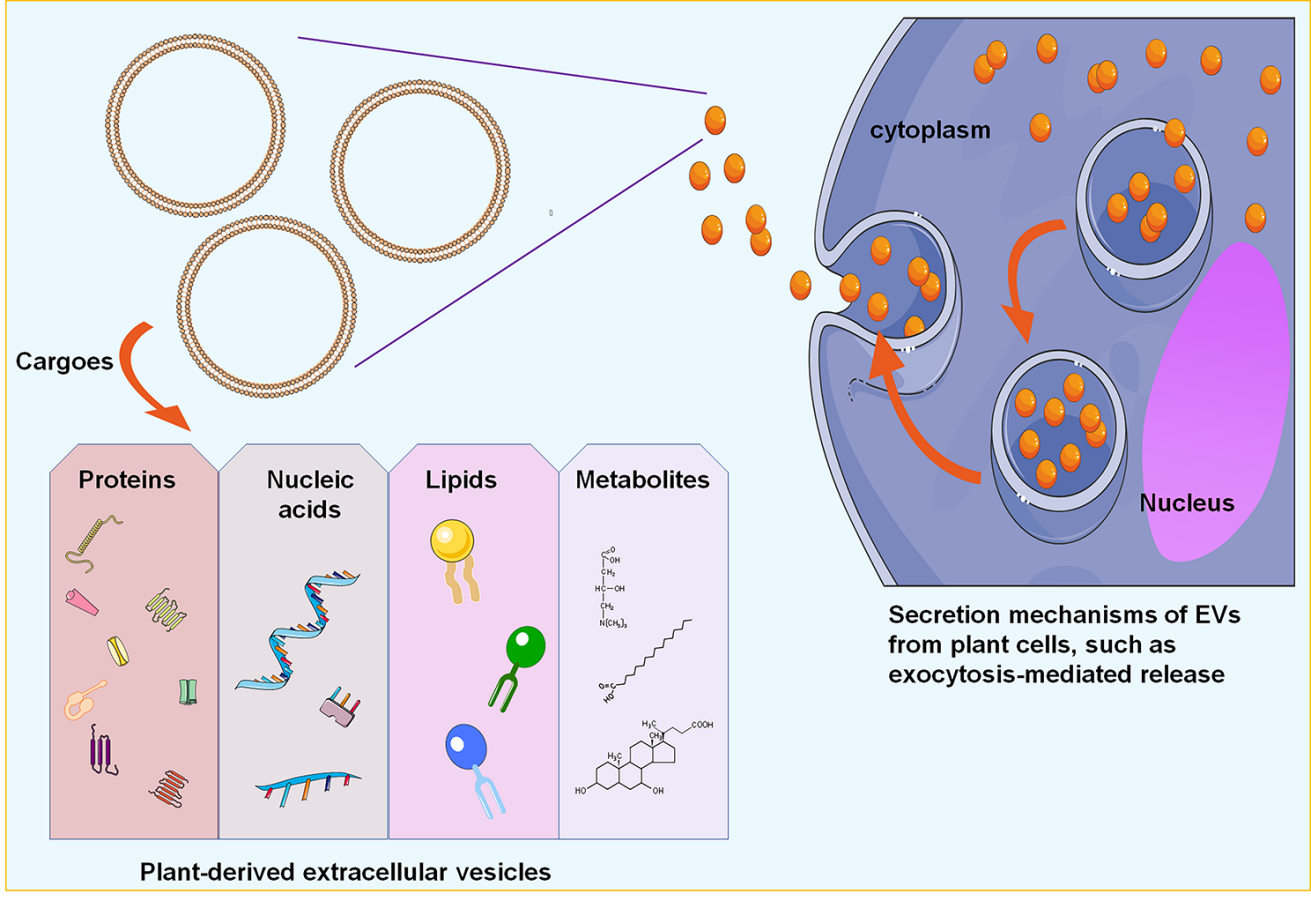
Schematic representations of PDEV structure and cargoes. PDEVs could transfer several cargoes to recipient cells, including small RNAs, proteins, lipids, and other pharmacologically active metabolites

PDEVs can transfer several cargoes to recipient cells and change the cellular phenotype, including nucleic acids, proteins, lipids, and other specific pharmacologically active substances [40] (Fig. 2). Several lipophilic secondary metabolites, such as alkaloids and curcuminoids, can be packaged into PDEVs and facilitate them to pass the membrane barrier [36]. Surprisingly, an ongoing work by Berger et al. [41] did not verify the secondary metabolites (vitamin C and naringenin) in the orange nanovesicles. These inconsistent findings may be due to the distinct scopes of nanovesicle-associated secondary metabolites in different plant species-derived EVs. Using label-free quantitative shotgun proteomics, Pocsfalvi et al. [9] found approximately 600–800 proteins in the citrus fruit juice sac cell-derived vesicles. Bioinformatic analysis revealed the important regulatory roles of these protein biocargo in multiple physiological processes, including vesicular trafficking, cellular metabolism, and cell growth. Subsequent studies supported the view that plant-derived microRNAs could be encapsulated in PDEVs, affecting the localization and stability of microRNAs [42–44]. The microRNAs transferred using PDEVs interfered with the pathophysiological processes of recipient cells by regulating their target genes [1]. Interestingly, these bioavailable cargoes encapsulated on PDEVs make them unique and powerful tools for health-beneficial purposes. In fact, without the need to reload other drugs, the PDEVs display natural clinical and pharmaceutical benefits [45]. For pharmaceutical application, the production of medicinal PDEVs did not exhibit any immunogenic or toxic effects on the host cells [46].

Besides the inherent biological activities, PDEVs can act as excellent nanovectors for the intercellular delivery of poorly soluble agents or therapeutic compounds as they may interfere with or potentialize their pharmacological activity. In addition, PDEVs are a potential source of desirable morphologies, feasible for large-scale production, inexpensive, and made of environmentally-friendly materials [47]. Man et al. (2021) extracted ginger-derived EVs (GDEVs) using ultrahigh-speed centrifugation. They found that the loading capacity of GDEVs strongly improved the lipid solubility of gingerol compounds, facilitating the intestinal absorption and transportation of gingerols [48]. Folic acid (FA)-positive ginger-derived EVs were used for the targeted delivery of survivin siRNA to human oral epidermoid carcinoma KB cells, leading to the downregulation of survivin and suppression of cell growth in vitro and in vivo [49]. The doxorubicin-containing PDEVs efficiently strengthened the cytotoxic effects of doxorubicin on the colon cancer cells, SW480. Meanwhile, other therapeutic agents, such as antisense oligonucleotides, were also encapsulated into PDEVs and successfully transported into human cells [50]. Collectively, these findings prove the value and potential of PDEVs as nanoscale vehicles for the delivery of therapeutic cargoes and offer a meaningful indication for the further use of PDEVs as a drug delivery system [51]. However, more investigations are still needed to exactly determine the potential of these nanoscale drug carriers to the next level for the benefit of patients in the future.

**Fig. 3:**
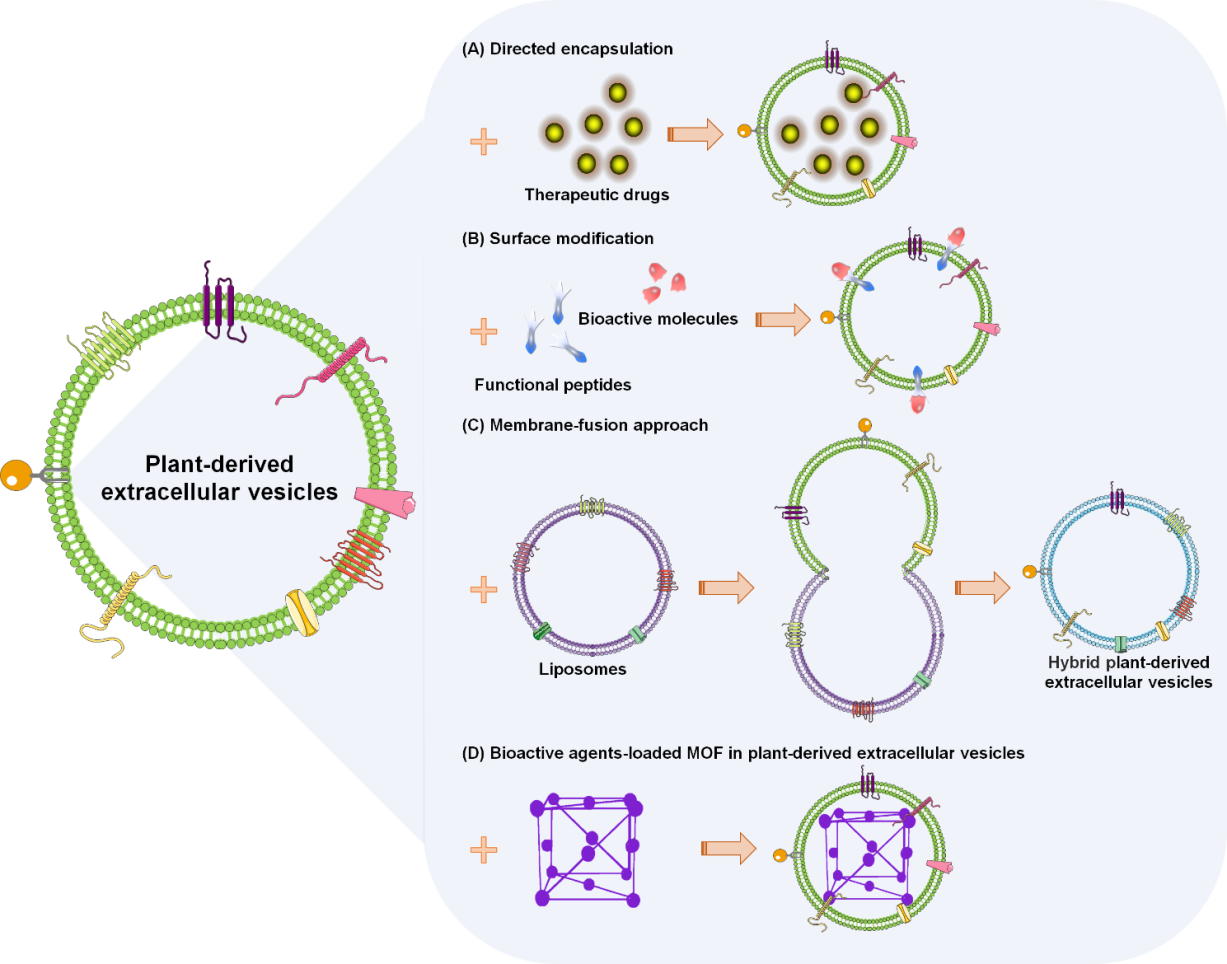
Modification techniques to facilitate PDEVs as the potential therapeutic nanocarriers. (A) Pharmacological particles directly encapsulated into the PDEVs. (B) Functional ligands effectively loaded onto the surfaces of EVs using a surface modification strategy. (C) Liposome-mediated membrane-fusion approach facilitating the design of advanced engineered PDEVs. (D) Bioactive agent-loaded MOF encapsulated into PDEVs, consequently prompting the targeted delivery of cargoes into recipient cells

Several studies focus on modification techniques to improve the drug delivery efficiency of PDEVs (Fig. 3). The pharmacological particles are directedly encapsulated into the PDEVs by internalization processes, such as phagocytosis or endocytosis, to be selectively taken up by the recipient cells [52]. Once inside the PDEVs, the therapeutic drugs are significantly transported to the inflammatory tumor sites, leading to the inhibition of tumor growth [53]. Tian et al. [54] developed another surface modification strategy, named the bio-orthogonal copper-free azide alkyne cyclo-addition. Using this heterobifunctional click chemistry, the functional ligands were effectively loaded onto the surfaces of EVs. For example, the cyclo(CRGDKGPDC), an integrin-specific iRGD peptide, promoted the conjugation efficiency of integrin αvβ3 on the EV membrane [55]. Recently, Sato et al. [56] fabricated an engineered hybrid EV by membrane fusion with various liposomes. Functional studies have demonstrated that the liposome-mediated membrane-fusion approach facilitates the design of advanced engineered EVs to deliver exogenous hydrophobic compounds across the tissue barriers [57]. Lastly, a review published in 2022 [58] indicated that the metal-organic frameworks (MOF), a synthetic porous functional material with excellent biocompatibility, could be regarded as a promising alternative for disease management. The bioactive compound-loaded MOF encapsulated into EVs prompted the delivery of cargoes into the targeted recipient cells [35, 59]. Thus, these modification methods provide novel opportunities for the significant advancement of PDEVs as appropriate nanodelivery systems.

### Properties of PDEVs for cancer research and treatment

Over the last few decades, studies exploring novel strategies for improving therapeutic efficacy have been a dominant topic in clinical cancer research. Nowadays, EVs derived from plants have been demonstrated to participate in regulating different biological functions and broadening cancer treatment strategies with several benefits [47].

The EVs isolated from *Kaempferia parviflora* (KPEVs) had the attractive ability to antagonize tumorigenesis. The engineered KPEVs were stably absorbed into the gastric adenocarcinoma (AGS) cells, leading to the suppression of cell viability in a dose-dependent manner [60]. Other interesting findings were the identification of exosome-like nanotherapeutics from tea flowers [61] and tea leaves [62]. The cellular experiments indicated strong cytotoxicity effects of these exosome-like nanotherapeutics against breast cancer cells in vivo and in vitro. Administration of exosome-like nanotherapeutics significantly stimulated the mitochondrial damage and reactive oxygen species (ROS) amplification, consequently triggering the apoptotic effect in 4T1 cells. Similarly, Yang and colleagues confirmed the functional roles of lemon juice-derived EVs (LDEVs) on ROS generation in cancer cells [63]. Using an innovative method, the electrophoretic technique combined with a 300 kDa cut-off dialysis bag, Yang’s group successfully isolated the LDEVs, and found that LDEVs were effectively internalized into the gastric cancer cells (AGS, BGC-823, and SGC-7901), causing improved ROS concentration and induction of cell apoptosis. In addition, the paper on “kinase-targeted cancer therapies” reviewed various protein kinases driving cancer progression, such as protein kinase AKT and extracellular-signal-regulated kinase (ERK) [64]. To further confirm these points, Stanly et al. [65] evaluated the effects of grapefruit-derived EVs (GFDEVs) on the AKT-ERK signaling pathway. They found that the administration of GFDEVs remarkably downregulated the activation of the AKT-ERK axis in different cancer cell lines, consequently causing cell cycle arrest and cell apoptosis. Overall, the data presented here strongly support the biomedical toxicity of PDEVs on cancer cells.

In addition, PDEVs are highlighted as novel agents that can be used to overcome the defection of conventional anti-cancer therapy. The potential utilization of these bioactive PDEVs in combination with conventional methods can open a new gateway for the treatment of cancer patients. Some conventional anti-cancer methods, such as radiotherapy, frequently cause excessive ROS generation, resulting in oxidative stress and side-effects in cancer patients [66]. Administration of the bitter melon-derived EVs (BMDEVs) scavenged the elevated mitochondrial ROS and maintained mitochondrial homeostasis, dominantly preventing radiation-induced cardiomyocyte injury and myocardial fibrosis [67]. Moreover, these BMDEVs exerted synergistic anti-cancer effects of 5-fluorouracil against oral squamous cell carcinoma [68, 69]. In vitro and in vivo analyses further demonstrated the sensitizing effect of BMDEVs on human cancer therapy [70]. The above findings demonstrate the clinical potential of PDEV-based strategies to enhance therapeutic efficacy.

Although a detailed understanding of PDEVs is not present, their applications in cancer research and treatment have attracted a great deal of attention. Notably, the PDEVs display good safety and biocompatibility for future clinical applications [71, 72]. Several groups consistently revealed that PDEVs displayed remarkable killing effects on cancer cells without significantly affecting normal cell growth [73, 74]. This view was further confirmed by Özkan and colleagues [75], who proved that the dermal fibroblast cells from a healthy person could remain unaffected upon treatment with garlic-derived EVs. Subsequently, the experimental animal models showed no significant weight loss in the mice after oral treatment with PDEVs [49]. In summary, these unique characteristics make PDEV-based strategies a profound candidate against cancer pathogenesis.

### Biomedical applications of PDEVs in anti-inflammatory response

Generally, the inflammatory response has been regarded as a phenomenon induced by imbalanced immune signaling. The improper control of this immune dysregulation can cause persistent inflammation, which may contribute to chronic or acute inflammatory diseases, threatening human health [76, 77]. To date, a series of experimental evidence has shown the great potential of PDEVs in the regulation of immune function and inflammatory responses in in vitro and in vivo models [78, 79]. The bioactive materials loaded on PDEVs, such as microRNAs, could be transferred into recipient cells and inhibit the SARS-CoV-2-induced inflammatory responses in the lung [80]. The EVs isolated from *Petasites japonicus* (PJ-EVs) showed increased activation of MAPK and NF-κB signaling, considerably inducing the maturation of murine dendritic cells and strengthening their antigen-presenting ability. Furthermore, treatment with PJ-EVs boosted the activation of Th1 T cells and cytotoxic T cells in inflammatory responses [81]. Another recent finding showed that nanovesicles derived from *Pueraria lobata*, an edible and medicinal herb, were well-taken up by mouse macrophages and facilitated M2 macrophage polarization. Through shifting M1 macrophages toward M2-like phenotypes, *Pueraria lobata*-derived EVs function as an attractive anti-inflammatory therapeutic biomaterial [82]. In addition, the roles of PDEVs in alleviating inflammatory-related diseases were directly demonstrated through the mulberry bark-derived EVs-mediated protection effect on intestinal epithelial cells in a mouse colitis model [83].

It is conceivable that the interaction between PDEVs and targeted cells can generate beneficial effects by mediating the secretion of multiple inflammatory factors. In an alcohol-induced chronic brain inflammation model, oat nanovesicles crossed the BBB and were preferentially taken up by the microglial cells. Oral administration of these oat nanovesicles decreased the secretion of pro-inflammatory cytokines, such as IL-6, IL-1β, and TNFα, from microglial cells and contributed to the prevention of ethanol-induced brain damage [84]. Similarly, the downregulated pro-inflammatory transcripts of IL-6, IL-1β, and COX-2 induced by pre-treatment with cabbage nanovesicles exhibited promising anti-inflammatory activities in human keratinocytes and fibroblasts [50]. In addition, PDEVs can be used to avoid inflammatory damage by promoting the upregulation of anti-inflammatory molecules, such as IL-9 and IL-10 [85]. The EVs from several fruits and vegetables, including grapes, grapefruit, ginger, and carrot, increased Wnt activation-mediated IL-10 secretion, providing beneficial effects for maintaining host cell homeostasis [45].

Oxidation-mediated inflammation has recently become an emerging research topic. Aging and other diseases are fundamentally caused by the imbalance between oxidation and anti-oxidation, which results in inflammatory injury and disease risks [86]. Using specific ELISA colorimetric assays, Logozzi’s group [87] identified high antioxidative content inside the PDEVs from fruit mixes, indicating their high level of antioxidant capacity. The strawberry-derived EVs were internalized by human mesenchymal stromal cells and prevented oxidative stress induced by hydrogen peroxide (H_2_O_2_) in a dose-dependent manner [88]. Similarly, the EVs from pomegranate juice showed an obvious effect to alleviate H_2_O_2_-induced oxidative damage [89]. The anti-inflammatory ability and health-promoting activity against oxidative stress of PDEVs have been proven to be mediated by up-regulating the expression of antioxidative molecules in the recipient cells, including nuclear factor erythroid 2-related factor 2 (Nrf2), heme oxygenase 1 (HO-1), and NAD(P)H quinone dehydrogenase 1 (NQO1) [90]. Through inhibiting the reduction of antioxidative molecules, PDEVs function as the prospective scavengers of free radicals, impairing their harmful cellular effects [91]. Taken together, this information may provide the basis for using PDEVs as biological antioxidant agents. In addition, it would be interesting to explore the underlying anti-oxidation mechanisms for more broad therapeutic applications in inflammatory diseases.

### Current findings and importance of PDEVs for skin-based therapy

Due to their highlighted therapeutic properties, the roles of PDEVs in dermatological conditions are of intense interest and raise some concern. Accumulating evidence has described the applications of PDEVs in the treatment of dermal diseases, such as cutaneous lesions, skin regeneration, and skin cancer [92, 93]. Thus, it is important to consider PDEVs as novel biotechnological skin protectants for maintaining normal skin function.

Every year, millions of individuals suffer from mutilating scarring and serious wounds that take much time to cure and impose a high infection risk. PDEV-based signaling has been recently proven to participate in the wound healing progress [94], supporting their attractive properties to facilitate wound-closure induction and acceleration. Savci et al. [95] demonstrated PDEVs as the prospective cell-free biomaterials for wound healing. In human epidermal keratinocyte HaCaT cells, the administration of GFDEVs remarkably promoted cell viability and reduced cellular ROS generation in a dose-dependent manner [95]. Moreover, the tube formation capabilities of human umbilical vein endothelial cells (HUVEC) were significantly increased after treatment with *Aloe saponaria*-derived EVs (AS-EVs) [96], suggesting the effective pro-angiogenesis activity of AS-EVs within the wound healing. After the injury, the ginseng-derived EVs (G-EVs) functioned as nanoplatforms for effective delivery of plant microRNAs into bone marrow-derived mesenchymal stem cells (BMSCs), consequently facilitating the BMSC neural differentiation and neural restoration surrounding the wound sites. G-EVs were also shown to stimulate neovascularization by increasing angiogenic factors, such as vascular endothelial growth factor [97]. All these biological progresses are significant for tissue regeneration and wound healing.

In addition, because of a few side-effects and high skin penetration, the natural sources could function as an alternative to chemotherapeutic agents [98, 99]. The spectrophotometric and biochemical approaches indicated that G-EVs could act as the immunopotentiator for altering the polarization of M2 macrophages, finally improving the anti-tumor immune response in melanoma-bearing mice [100]. The EVs extracted from *Dendropanax morbifera* were uptaken by B16BL6 melanoma cells, resulting in reduced cellular melanin concentration and increased whitening effect by impairing tyrosinase-related signaling. Notably, treatment with *Dendropanax morbifer*-derived EVs induced nonsignificant cytotoxicity to healthy human skin tissues [101].

For better bio-therapeutic purposes, PDEVs can be engineered to contain specific pharmaceutical compounds or used for targeted transport by labeling with certain surface biomarkers [102, 103]. For example, in 2020, Yepes-Molina et al. [104] explored the biological potential of broccoli-derived EVs (BDEVs) as agent delivery nanoplatforms. The membrane vesicle-encapsulated fluorescent products were notably detected in keratinocyte skin cells, suggesting BDEVs as nanosized technology for the transdermal delivery of drugs. Furthermore, to enhance skin permeation of plasmid DNA (pDNA) into melanoma tissues, Niu’s group established the cell-penetrating peptide and cationic poly(ethyleneimine) conjugated pDNA-loaded PDEVs. They found that these functional peptide-conjugated PDEVs were highly efficient in facilitating transdermal pDNA delivery into skin melanomas [105].

### PDEV-based therapy in clinical trials

In the last couple of decades, many studies have extended the understanding of the scientific community about the functional advantages of PDEVs, highlighting possible novel insights in biomedicine for the treatment of human diseases. In this regard, several clinical studies on the beneficial activities of PDEVs for clinical management have been currently registered in the ClinicalTrials.gov database [106] (Table 3). In particular, the properties of EVs isolated from many kinds of fruits and their ability as a delivery vehicle to increase the bioavailability of oral curcumin are being tested on human colon cancer patients (NCT01294072) [107]. In a subsequent study, Zhang et al. [108] aimed to evaluate the great prospects of GDEVs as natural anti-inflammatory agents for irritable bowel disease (NCT04879810). This trial concluded that GDEVs were likely reliable therapeutic nanoparticles for effectively preventing inflammatory bowel disease. Another completed trial confirmed the anti-inflammatory activity of grape-derived EVs (NCT01668849) [107]. Additionally, they proved the important roles of grape-derived EVs in preventing some side-effects induced by chemoradiation treatment in head and neck cancer patients, such as oral mucositis pain. Finally, an exploratory trial was designed to evaluate the effectiveness of EVs isolated from ginger or aloe plants in the treatment of patients with polycystic ovarian syndrome (NCT03493984); however, this meaningful study has not been approved so far. Therefore, considering the above-mentioned promising advantages [109, 110], the researchers should conduct more preclinical and clinical trials to determine the bioactivities of PDEVs in humans and define their minimum dosage in further studies.

**Table 3:** The clinical trials of PDEVs and their great potential for biomedicine research

Identifier	Plants	Status	Diseases	Functions	Refs
NCT01294072	Fruits	Recruiting	Colon cancer	Deliver curcumin to cancer tissues after oral administration	(107)
NCT04879810	Ginger	Completed	Irritable bowel disease	Anti-inflammatory effects	(108)
NCT01668849	Grape	Completed	Head and neck cancer	Reducing oral mucositis during radiation and chemotherapy	(107)
NCT03493984	Ginger or aloe plants	Withdrawn	Polycystic ovary syndrome	Mitigating insulin resistance; Anti-Inflammation	/

### Critical thinking and future outlooks

It is well established that PDEVs could act as important information conveyers between donor and recipient cells by transporting various bioactive cargoes, including proteins, nucleic acids, and other therapeutic agents. Although multiple omics techniques exploring their contents are increasing, the lack of specific biomarkers still results in the poor characterization of PDEVs and remains a major challenge [111]. The development of new computational algorithms to analyze the PDEV-associated omics data is a significant requirement. Recently, the characteristics of nanoparticles from plants have been annotated in FoodEVs from FAO/INFOODS database (https://www.fao.org/infoods/infoods/tables-and-databases/faoinfoods-databases/en/) [37]. Beyond doubt, the emerging studies on PDEVs have already revolutionized our knowledge of intercellular communication. Furthermore, PDEVs display the capacity to re-engineer themselves with specific biomarkers or compounds for precise therapy. However, there are still several unresolved concerns governing the extraction and function of PDEVs.

**Fig. 4:**
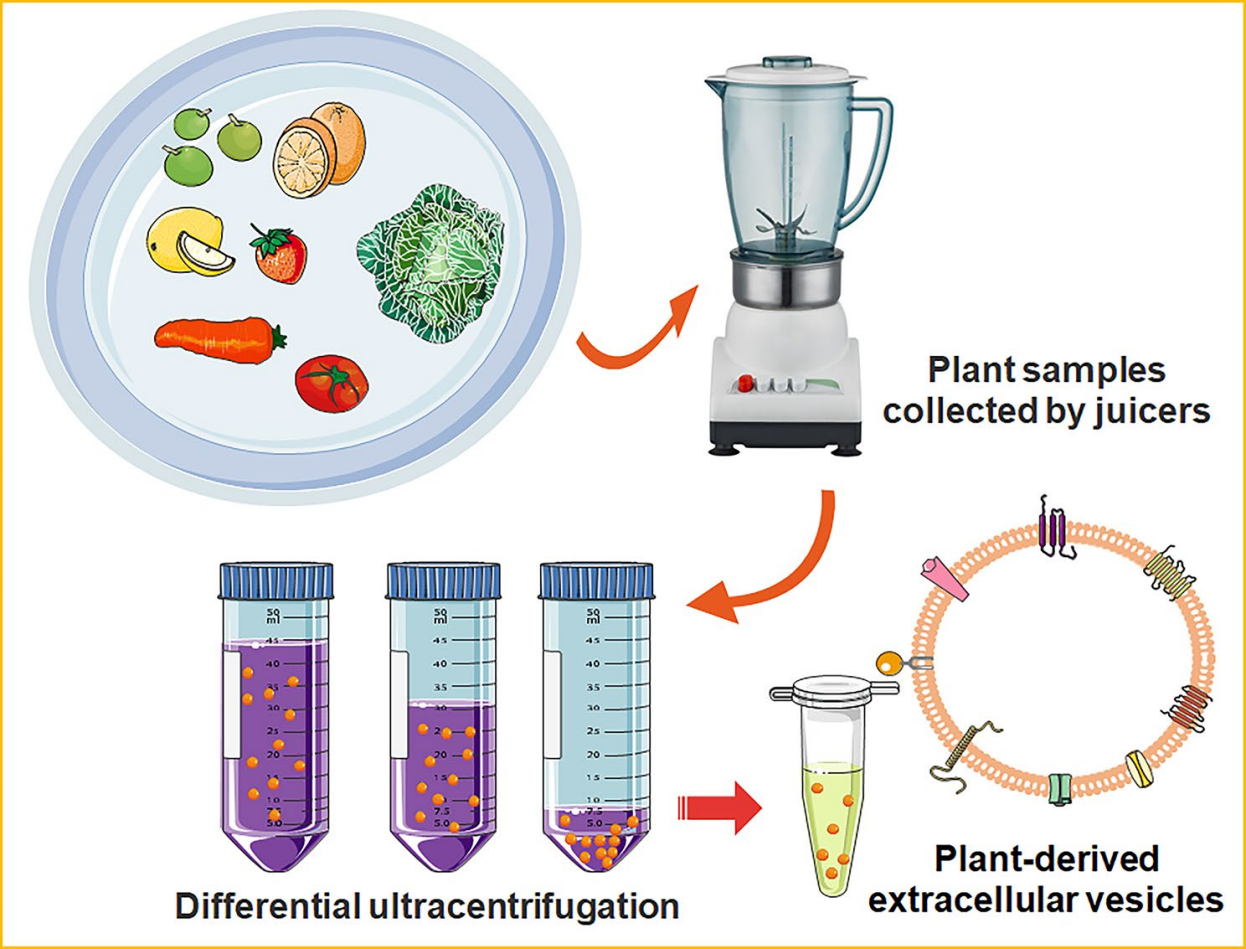
Representative steps of ultracentrifugation-based strategies for PDEV extraction

Currently, technological advancements have allowed researchers to isolate and characterize EVs. Several methods, such as filtration plus centrifugation, polymer-based precipitation, microfluidics technologies and immunoaffinity capture-based isolation, are frequently utilized for mammal-derived EVs but not for PDEVs [33, 103]. These techniques require different sample pre-processing procedures, and produce mammalian EVs of varying quality and purity. The EVs from cell culture media or body fluids are frequently subjected to multiple centrifugal procedures [112]. It’s worth noting that these centrifugation steps may be different depending on the experimental design, sample properties, and downstream analysis [113]. The polymer-based precipitation is a strategy employed to isolate EVs from the biofluids by altering their solubility. Using the commercial ExoQuick-TC kit, polymer-based precipitation could yield higher quantities of exosomal cargoes than the centrifugation [114]. Alternative method for isolation of mammalian EVs is a microfluidic exosome isolation and detection system (EXID system), which could incorporate the exosome capture and biomarker labelling on a microfluidic chip [115]. This integrated system enables one-stop capture, isolation and detection of exosomes from cancer cells and peripheral blood samples. However, the purification methods for PDEVs are still waiting to be fully tapped. To date, the most traditionally used techniques for PDEV extraction are the different centrifugal-based methods. In 2009, the Regente lab initially developed the vacuum infiltration-centrifugation procedure and applied this technology to successfully separate the sunflower seed-derived EVs [13]. Later, multiple research groups used differential ultracentrifugation to separate and purify EVs from plant tissues [116–118]. A recent review by Urzì O, Raimondo S, and Alessandro R succinctly summarized the representative steps of ultracentrifugal methods for PDEV extraction [111] (Fig. 4). In brief, after the tissues are manually squeezed by juicers, low-speed centrifugation at 500–3000 g for about 30 min was initially used to remove large particles and plant fibers. Next, intermediate-speed centrifugation, about 2000–10,000 g for 30 min, was used to remove the cell fragments and organelles. At last, high-speed centrifugation at 100,000–150,000 g for about 2 h was used to acquire the EV pellets. Although specialized equipment and much time are required, these ultracentrifugation-based strategies have been defined by different studies as the well-established gold standard for PDEV preparation [119]. In addition, some handy and rapid isolation methods have been established to facilitate future biochemical studies and downstream applications of PDEVs. The project of Jackson’s group was to evaluate a rapid hydrophobic interaction chromatography (HIC)-based capillary-channeled polymer (C-CP) tip isolation strategy for PDEV isolation. This HIC-based C-CP tip successfully obtains the desirable integrity and yield of PDEVs and is less time-consuming and low-cost [120]. Another efficient method for PDEV preparation, electrophoresis combined with a 300 kDa cut-off dialysis bag, has also been demonstrated to be time-saving [63]. Şahin et al. [121] utilized a commercial kit, Exo-spin™ Exosome Purification Kit, to isolate the homogenous and stable PDEVs from wheat grass juice. Although efficacious, most of these processes depend on specialized instruments or lack standard protocols, consequently representing a limitation for their clinical applications [106]. The method using polyethylene glycol (PEG) could effectively prevent *Nicotiana tabacum*-derived vesicles from forming the aggregates [122]. In the future, there is an urgent need to develop the standardization of qualitative and quantitative procedures to better achieve the successful commercialization of PDEVs in the field of translational medicine.

It should be noted that environmental conditions critically affect the stability and biological activities of PDEVs. Moreover, the intrinsic properties of isolated PDEVs are subject to change with some physical parameters, including pH values, temperature, and other processing factors [3, 15]. Under a simulated stomach condition, the PDEVs have the potential to resist gastric digestion and maintain high stability [60]. Unfortunately, at the moment, few studies have specifically investigated the effect of the environment on the structural integrity and bioactivity of PDEVs [109]. A recent study by Berger’s group showed that the altered morphology of PDEVs was found in the unpasteurized juice prepared by industrial processes. Furthermore, no PDEVs were detected in the concentrated orange juice [41]. By comparing the quantity and quality of PDEVs isolated from organic farms or conventional farms, Logozzi et al. [87] found that the former materials resulted in greater yield and higher anti-oxidant capacity. Thus, various environmental factors should be well considered for the plant sample collection and PDEV extraction.

The pathogen invasion generally induces infection and damages plant tissues. Phytoviruses are widely observed in animals, humans, as well as other environmental substances [123]. Independent scholars successfully confirmed the existence of phytovirus infection-associated molecules in the plant resource-derived nanovesicles [124, 125]. Moreover, they recognized several similar physical features between virus particles and PDEVs, such as particle size. In addition, plant viruses present in plant materials were frequently co-extracted with PDEVs [126], suggesting viral contamination in plant nanovesicle samples. Thus, it is imperative to develop novel platforms and strategies to remove virus particles from PDEV isolates and overcome virus contamination. Accordingly, with the help of the sucrose- or iodixanol-based density gradient ultracentrifugation technique (DGUC), Mammadova and colleagues effectively separated PDEVs from viral particles in tomato homogenate [126]. This has motivated the exploration of developing more promising technologies to purify PDEVs without possible virus contamination.

## Conclusions

In conclusion, emerging evidence from preclinical and clinical studies has shed light on the biomedical application potential of natural PDEV-based strategies for pharmacogenetic discovery and validation. The molecular, biological, genetic, and pharmacological technologies suggest that natural and endogenic PDEVs have many attractive advantages, especially high stability, intrinsic bioactivity, and easy absorption by the recipient cells. Furthermore, numerous bioactive cargoes with pharmaceutical interest were demonstrated on PDEV isolates. As a promising drug delivery system, PDEVs can increase the sensitivity of drugs against numerous pathologies and concomitantly reduce their toxic side-effects. However, the underlying molecular mechanisms and biological factors driving the functions of PDEVs in diseases remain to be defined. The already-existing information offers fascinating and promising insights into the potential benefits of PDEVs for their medical translation in the future.

## Data Availability

The data used to support this review are included within the article.

## Abbreviations

PDEVs: Plant-derived extracellular vesicles
EVs: Extracellular vesicles
BBB: Blood-brain barrier
GDEVs: Ginger-derived EVs
FA: Folic acid
MOF: Metal-organic frameworks
KPEVs: EVs isolated from *Kaempferia parviflora*
ROS: Reactive oxygen species
LDEVs: Lemon juice-derived EVs
ERK: Extracellular-signal-regulated kinase
GFDEVs: Grapefruit-derived EVs
BMDEVs: Bitter melon-derived EVs
PJ-EVs: EVs isolated from *Petasites japonicus*
H_2_O_2_: Hydrogen peroxide
Nrf2: Nuclear factor erythroid 2-related factor 2
HO-1: Heme oxygenase 1
NQO1: NAD(P)H quinone dehydrogenase 1
BMSCs: Bone marrow-derived mesenchymal stem cells
AS-EVs: *Aloe saponaria*-derived EVs
G-EVs: Ginseng-derived EVs
HUVEC: Human umbilical vein endothelial cells
BDEVs: Broccoli-derived EVs
pDNA: Plasmid DNA
HIC: Hydrophobic interaction chromatography
C-CP: Capillary-channeled polymer
DGUC: Density gradient ultracentrifugation technique
